# 

**Published:** 1867-11

**Authors:** 


### CONTENTS.

### ORIGINAL COMMUNICATIONS.

PAGE.

ARTICLE I.—Acute Glaucoma of both Eyes Successfully treated by Paracentesis of the Cornea. By W. F. Westmoreland, M. D., Professor of Surgery in the Atlanta Medical College ........ 373

ARTICLE II.—Gun-Shot Wound—Delirium Tremens, Administration of Chloroform in, etc. By L. H. Orme, M. D., Atlanta, Ga ........ 376

ARTICLE III.—Extraction of a Large Stone from the Urethra of a Boy three years old. By J. F. M. Davis, of Gum Springs, Va ........ 379

ARTICLE IV.—A Case of Doublets—Uterine Inertia—Alarming Sequences. By E. L. Connally, M. D., Albany, Ga ........ 381

### SELECTIONS.

Stricture of the Œsophagus ........ 388

Papers on Skin Diseases ........ 390

### EDITORIAL AND MISCELLANEOUS.

Causation of Fever ........ 396

European Correspondence ........ 407

Bibliographical ........ 409

Miscellaneous Items ........ 411

